# Pulmonary embolism in medical-surgical ICU patients

**DOI:** 10.1186/cc9439

**Published:** 2011-03-11

**Authors:** D Heels-Ansdell, N Zytaruk, M Meade, S Mehta, R Hall, R Zarychanski, M Rocha, W Lim, F Lamontagne, L McIntyre, P Dodek, S Vallance, A Davies, DJ Cooper, DJ Cook

**Affiliations:** 1 McMaster University, Hamilton, Canada; 2 Mount Sinai Hospital, Toronto, Canada; 3 Capital Health - QEII, Halifax, Canada; 4 Cancer Care Manatoba, Winnipeg, Canada; 5 Sherbrooke Hospital, Quebec, Canada; 6 Ottawa Health Research Institute, Ottawa, Canada; 7 St Paul's Hospital, Vancouver, Canada; 8 Alfred Hospital, Melbourne, Australia

## Introduction

Pulmonary embolism (PE) is a feared complication of critical illness. PE is difficult to diagnose during critical illness due to the nonspecificity of signs and symptoms and low index of suspicion in practice. Our objective was to examine the antecedent characteristics and hospital course of patients who were diagnosed with PE during critical illness in the context of an international trial of thromboprophylaxis (NCT00182143).

## Methods

Research coordinators documented all clinical, laboratory, radiologic and autopsy criteria relevant to PE, which was a secondary outcome for this multicenter trial. Patients with a possible PE were adjudicated in quadruplicate; those considered possible, probable or definite PE were considered in this analysis. PEs were considered clinically suspected if the ICU team conducted tests seeking a diagnosis; otherwise, they were incidental.

## Results

In 3,659 patients, PE was clinically suspected in most patients who were diagnosed with a prevalent PE at ICU admission (12/14, 85.7%) or incident over the course of the ICU stay (57/64, 89.1%). Among 64 patients who developed a PE, only three (4.7%) had prehospital DVT or PE. Within the index hospitalization, before or after the PE diagnosis, additional acute deep venous thromboses occurred at any site in 27 (42.2%) patients with PE. Patients without PE compared with those with PE appear to have a shorter duration of ventilation (median, interquartile range) (5 (2, 11) days vs. 12 days (5.5, 20.5), *P *< 0.001), duration of ICU stay (9 (6, 16) days vs. 20.5 (13, 35), *P *< 0.001), and hospital stay (21 (13, 40) days vs. 35 (21.5, 58.5), *P *< 0.001), and a lower ICU mortality (15.2% vs. 31.8%, *P *= 0.005) and hospital mortality (22.8% vs. 31.3%, *P *= 0.13).

## Conclusions

The majority of PEs in these medical-surgical ICU patients were clinically suspected rather than incidental findings. More than one-half of the PEs developed in the absence of leg or other venous thromboses; in some cases, additional venous thromboses post-dated rather than pre-dated the PE. PE was associated with significantly increased morbidity and mortality in this ICU population.

